# Association of Rare Earth Elements with Passive Smoking among Housewives in Shanxi Province, China

**DOI:** 10.3390/ijerph19010559

**Published:** 2022-01-04

**Authors:** Jigen Na, Huiting Chen, Hang An, Nan Li, Lailai Yan, Rongwei Ye, Zhiwen Li

**Affiliations:** 1 Key Laboratory of Reproductive Health, National Health Commission of the People’s Republic of China, Institute of Reproductive and Child Health, Peking University, Beijing 100191, China; najigen@bjmu.edu.cn (J.N.); htchen1999@163.com (H.C.); anhang@bjmu.edu.cn (H.A.); yerw@bjmu.edu.cn (R.Y.); 2 Department of Epidemiology and Biostatistics, School of Public Health, Peking University, Beijing 100191, China; 3 Department of Laboratorial Science and Technology, School of Public Health, Peking University, Beijing 100191, China; yll@bjmu.edu.cn

**Keywords:** rare earth elements, emerging contaminant, passive smoking, biomarker, housewives

## Abstract

Background: Rare earth elements (REEs) are emerging contaminants. Previous studies reported the association between REEs and active smoking, but little is known about the effects of passive smoking on this condition. In China, female passive smoking is widespread, particularly in rural areas. Objective: This study aimed to estimate the relationship between REEs accumulation and passive smoking among rural housewives. Methods: We recruited 385 subjects in Shanxi Province of northern China, of whom 117 housewives were exposed to passive smoking, and 268 were not. We analyzed 15 REEs in the hair of housewives with ICP–MS, including lanthanum, cerium, praseodymium, neodymium, samarium, europium, gadolinium, terbium, dysprosium, holmium, erbium, thulium, ytterbium, lutetium, and yttrium. Results: The results indicated higher levels of 14 REEs except for Sm in both the univariate and adjusted models among the housewives exposed to passive smoking. The increasing linear trend of adjusted odds ratios of 15 REEs supported their association. The Bayesian kernel machine regression (BKMR) models showed that 15 REEs had a significant overall effect, and Eu had a single-exposure effect with passive smoking. Conclusion: We concluded that passive smoking might be associated with increased exposure to REEs among rural housewives.

## 1. Introduction

Passive smoking has been commonly observed in multinomial categorical distributions pertaining to smoking status [1]. Many studies have shown that women have a lower active smoking rate but a higher risk of exposure to environmental tobacco smoke than men [2,3]. Women may be less cognizant of the damage caused by passive smoking and therefore may be less inclined to avoid smokers [4]. Women are the major recipient of secondhand smoke in China, and nearly 70% of rural women are exposed to passive smoking [3]. A survey in 2013 showed that the rate of passive smoking was 40.4% among women in central and Western China (including Shanxi Province) [5]. The majority of women, particularly those in rural areas, remain unaware of the detrimental health effects of passive smoking. Secondhand smoke has been associated with female sexual dysfunction, cervical carcinoma, breast cancer, and other gynecological diseases [6,7,8]. For pregnant women, passive smoking is a risk factor for adverse birth outcomes [9,10]. Although some studies have revealed that tobacco smoke has more than 60 kinds of hazardous substances [11], the degree of exposure to some pollutants through passive smoking is still unclear.

Rare earth elements (REEs), a class of trace metals with similar physicochemical properties, have attracted increasing attention due to their applicability in fields such as agriculture (e.g., lanthanum and cerium in corn and mungbean cultivation) and industry [12,13,14]. China also has rich, rare earth mine resources, and it has been found that REEs can enter the human body through air, food, and other sources [15,16]. Many studies have shown that REEs as emerging contaminants may lead to health impacts. Lanthanum (La) and neodymium (Nd) in the umbilical cord of pregnant women are risk factors for orofacial clefts [17]. Prenatal exposure to REEs might also overweigh an added risk of premature rupture of membranes [18]. In addition, it has been reported that all kinds of REEs can be detected in tobacco and rolling paper [19], and higher concentrations of lanthanum and cerium can be found in the indoor air of smokers’ homes and the hotels with environmental tobacco smoke [20], which both indicate that cigarettes may be a source of REEs exposure. A previous study reported the concentrations of serum REE among active cigarette smokers [21]; however, the association between REEs and passive smoking is still unclear.

We previously found that passive smoking had significant associations with rural housewives’ hypertension and influenza-like illness in Shanxi Province of China [4,22], and 15 REEs might bring negative effects to their health [23]. Therefore, these findings enable us to further explore the relationship between 15 REEs’ concentrations in housewives’ hair and passive smoking.

## 2. Methods

### 2.1. Study Design and Population

We conducted a cross-sectional study in Pingding County Hospital in Shanxi Province of northern China from August 2012 to May 2013, with the aim of assessing the effects of indoor air pollution on the health of local women. Detailed information on the study protocol was reported elsewhere [4]. The women were invited to participate in our study if they were (1) nonimmigrant residential status of Pingding County; (2) had no marked change in their living situation in the past 10 years; and (3) were aged 30 or over. In addition, our subjects were recruited in the countryside far from the REE mining area and mainly engaged in housework.

The characteristics of these recruited subjects were collected by a questionnaire, mainly including passive smoking status, age, occupation, education, menopause status, and the frequencies of hard drink (“baijiu” in Chinese) and weak drink (beer, red wine, or rice wine) intake, and physical activity. Physical examinations, such as measurements of height and weight, were performed by local physicians according to standard protocols. BMI (kg/m^2^) was computed as the value of weight divided by the square of height to be a possible confounding variable. This study was approved by the Institutional Review Board of Peking University. All participants were informed and signed the consent form.

Passive smoking in this study was defined as nonsmoking women who continued to stay in an environment with tobacco burning for more than 30 min. Excluding subjects with ongoing active smoking, the remaining subjects exposed to passive smoking at least once per week were considered the exposed group; otherwise, they were considered the control group.

### 2.2. Sample Collection and Analysis

Each included subject provided a strand of hair sample for analysis, which was sheared as near as possible to the scalp from the occipital part of the head. These samples were kept in closed labeled polyethylene zip-lock bags until analysis in the laboratory. The specific analysis method was detailed in our previous study [24]. In brief, we assumed that the growth rate of hair was 1 cm per month; thus, hair close to 24-cm long could indicate a woman’s 2-year exposure to REEs. Then, the eligible hair was cut into 1-cm segments and weighed to 80 mg. These samples and the blank vials were sequentially washed with 1 mL Triton X-100 (Sigma-Aldrich, St. Louis, MO, USA) 1 time, 1 mL deionized water 3 times, and 1 mL acetone (J.T. Baker^®^, Center Valley, PA, USA) 3 times. All of the above washes were under vortex for 5 min each time. Subsequently, these samples were digested with 1 mL nitric acid in a 15-mL quartz digestion tube in a microwave digester (Ultra WAVE, Milestone, Italy) for 50 min. The concentrations of REEs were detected using inductively coupled plasma–mass spectrometry (ICP–MS; ELAN DRC Ⅱ, PerkinElmer, Billerica, MA, USA).

The 15 REEs and their limits of detection (LODs, ng/mL) were as follows: lanthanum (La), 0.006; cerium (Ce), 0.0004; praseodymium (Pr), 0.0001; neodymium (Nd), 0.003; samarium (Sm), 0.0004; europium (Eu), 0.001; gadolinium (Gd), 0.002; terbium (Tb), 0.0004; dysprosium (Dy), 0.001; holmium (Ho), 0.002; erbium (Er), 0.012; thulium (Tm), 0.007; ytterbium (Yb), 0.001; lutetium (Lu), 0.0001; and yttrium (Y), 0.001. Each sample corresponded to three procedural blanks and one reagent blank, and the concentration was calculated by subtracting the means of the corresponding blanks from the detected concentration. The analysis results were finally converted to values in the unit of ng/g hair. When lowered to a certain blank or the LOD, the REE content was considered a value of zero.

### 2.3. Statistical Analysis

The demographic information between the exposed and control groups was shown as the mean ± standard deviation (SD) and tested using the *t*-test for normal-distributed continuous variables or as the number (percentage) and tested using the chi-square test for categorical variables. Since the concentrations of REEs were not normal-distributed, they were described as the median (interquartile range (IQR)), and the differences between the two groups were determined using the Wilcoxon signed-rank test. The correlation of any two REEs was expressed by Spearman’s rank correlation coefficient.

We performed both univariate and adjusted logistic regression analyses to study the associations between the single element and passive smoking. Because the possible dose thresholds of REEs in hair were unavailable, we bisected the concentrations of the individual REEs into two levels according to their respective median. Then, we calculated the odds ratio (OR) with its 95% confidence interval (CI) to estimate their association. The covariates involved age, BMI, occupation, education, menopause status, and the frequencies of wine drinking, weak drink intake, and physical activity. In addition, we examined the linear trend of the adjusted ORs under four different concentration levels, which were cut off by the corresponding 25th percentile (P_25_), 50th percentile (P_50_), and 75th percentile (P_75_).

**Figure A1:**
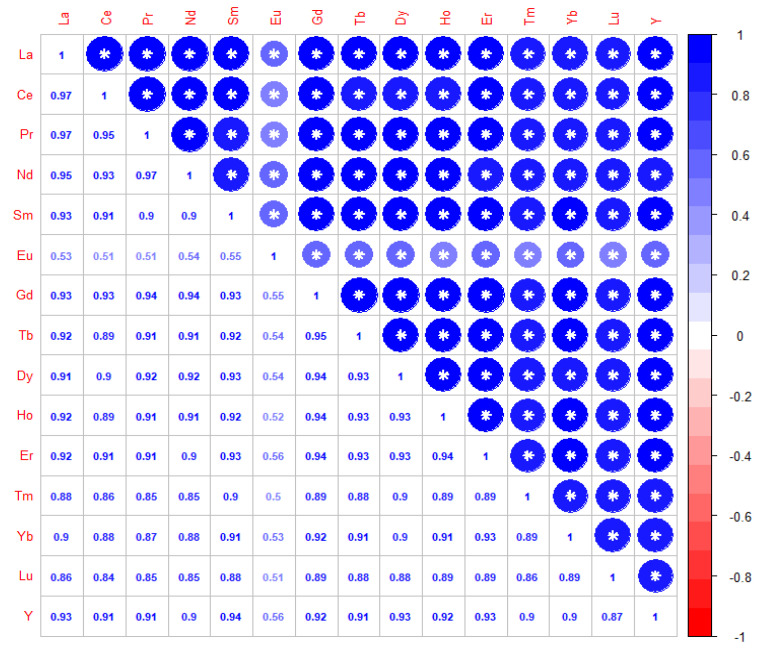
The plot of the spearman’s correlation matrix of 15 REEs. The concentrations of any two REEs in hair indicated significant correlations.

Considering that 15 REEs may have multicollinearity (for significant correlations between any two REEs, see in Appendix A Figure A1), the conventional logistic regression cannot meet the simultaneous analysis of multiple exposures. As a consequence, we implemented the Bayesian kernel machine regression (BKMR) model to evaluate the mixed risk of 15 REEs, which is an emerging approach for estimating the joint effect of the simultaneous mixture of multipollutant [25] and has been widely applied in other similar studies [26]. The principle of BMKR is based on a model of Y = h(Z_i_) + βX_i_ + e_i_. In the above equation, Y is the dependent variable, h(Z_i_) is the exposure-response function of independent variables, βX_i_ is the covariate with its effect, and e_i_ means residual values. BKMR can visualize both cumulative and single-exposure effects. The cumulative effect is defined as the change of the posterior mean estimate (and its 95% CI) of the exposure-response function, which is compared at the different percentiles to at the P_50_ of mixture components. The single-exposure effect is the value calculated by comparing the posterior mean estimate when a specific exposure is at its P_75_ to the one at its P_25_ at the same time when the other exposures are fixed at their P_25_, P_50_, or P_75_. Because the variable Y (passive smoking) was binary, we used the probit-BKMR to calculate the crude and adjusted models. The involved covariates were the same as in the adjusted logistic regression.

All of the data were analyzed with R software (version 4.0.2; R Development Core Team), and the BKMR was supported by the R package bkmr (version 0.2.0). Two-sided probability was used in all statistical tests. The criterion for significance was *p* value <0.05.

## 3. Results

### 3.1. Study Population Characteristics

A total of 405 housewives with 398 independent hair samples were involved in our study. After excluding 13 housewives with active smoking, 385 cases were finally analyzed, which included 117 in the exposed group and 268 in the control group. The characteristics of these housewives are shown in Table 1. No significant difference was observed except in the frequency of physical activity. Housewives exposed to passive smoking had more physical activities than the controls. The overall similar characteristics indicated that the two groups were comparable.

### 3.2. Association of Hair REEs with Passive Smoking

We found that rather than the controls, the exposed group had significantly higher concentrations of 15 REEs in hair, and the overall order of medians was Ce > La > Nd > Y > Pr > Gd > Sm > Dy > Eu > Er > Yb > Tb > Ho > Lu = Tm. The concentrations of REEs as well as each detection rate (DR) are shown in Table 2.

The logistic regression results for dichotomized variables of 15 REEs are shown in Table 3, which suggested that except for Sm, the other 14 REEs had significant associations with passive smoking regardless of the univariate or adjusted model. The univariate and adjusted ORs of 14 REEs were all between 1 and 3, which denoted housewives with passive smoking had 1–3 times the risk of being exposed to the higher concentrations of 14 REEs than those without any passive smoking.

**Figure 1:**
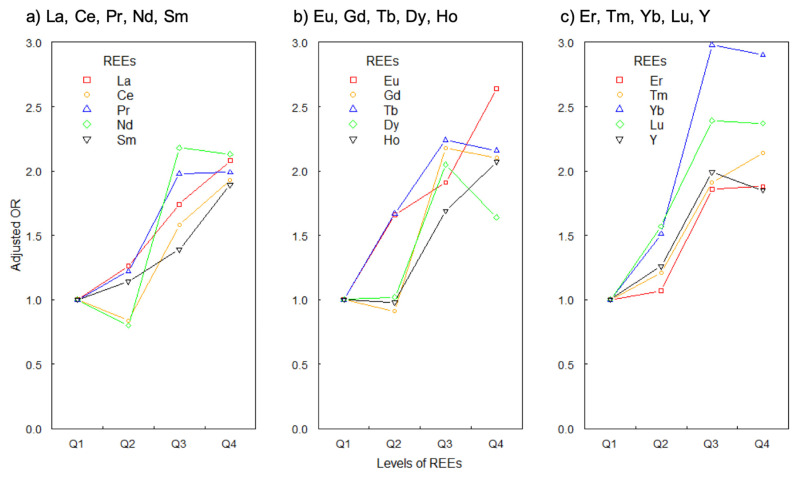
The adjusted ORs of 15 REEs in quartile intervals and their linear trends. (**a**–**c**) jointly showed significant linear trends of 15 REEs’ adjusted ORs (all the *p*-trend values were < 0.05).

When REEs were divided into four categories, the result of the trend test showed significant linear trends of adjusted ORs on the whole in all 15 REEs, including Sm, which indicated that increasing levels of each of 15 REEs had an association with passive smoking (*p* < 0.05). We used the line chart to express the trend (see Figure 1).

### 3.3. Bayes Kernel Machine Regression

**Figure A2:**
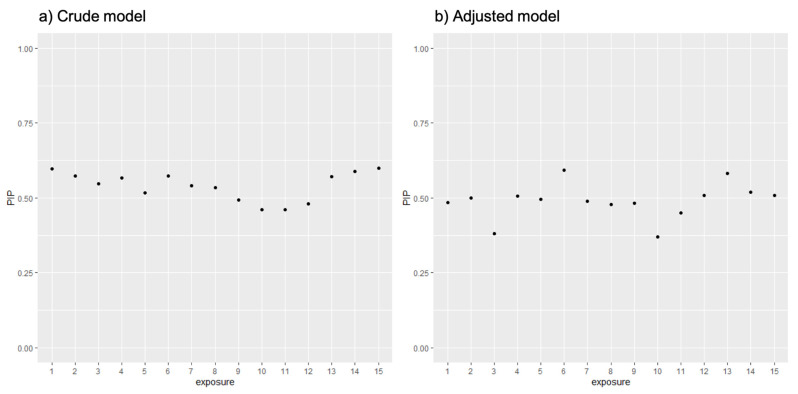
The plot of posterior inclusion probability of 15 REEs in BKMR model. (**a**,**b**) showed the posterior inclusion probability (PIP) in the crude model and the adjusted model, respectively. The PIPs of 15 REEs were all approximately 50% in both (**a**,**b**). It might indicate that these REEs were of similar importance in BKMR models and should be analyzed together.

Spearman’s rank correlation analysis presented significant correlations between any two REEs (see Appendix A Figure A1). We therefore conducted BKMR models to avoid the collinearities among 15 REEs. The models reported the posterior inclusion probability (PIP), and the PIPs of 15 REEs were all approximately 50%, which indicated that these REEs were of similar importance in the models (see Appendix A Figure A2).

**Figure 2:**
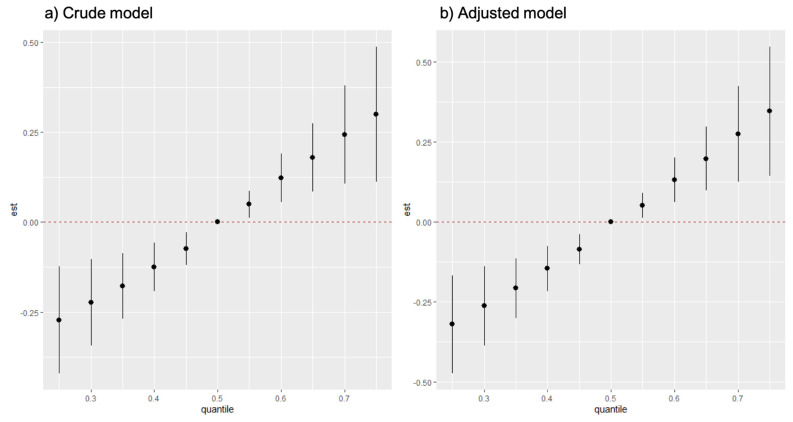
The cumulative effect of 15 REEs in the BKMR model. (**a**,**b**) showed the overall effect of the crude model and the adjusted model, respectively. The P_50_ level of the mixture showed significant differences from other levels and showed linear trends as the levels increased in both (**a**,**b**).

The change in cumulative effects is shown in Figure 2. As the levels of the mixture increased, so did the cumulative effect. When compared to the P_50_ of the mixture, the cumulative effects and their 95% CI at different levels were away from zero in both crude and adjusted models. The results showed that the increase in the 15 REEs as a whole was associated with passive smoking status.

**Figure 3:**
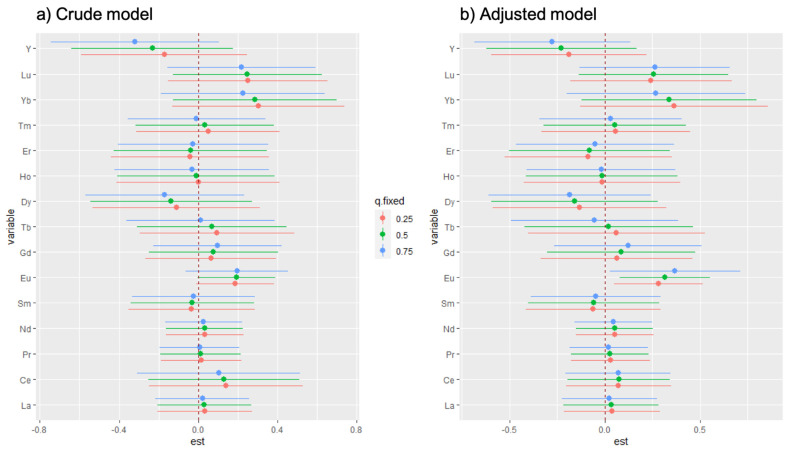
Single-exposure effect of 15 REEs in the BKMR model. (**a**,**b**) showed the contribution of certain REEs for the mixture exposure in the crude model and the adjusted model, respectively. (**a**) did not indicate any significant single-exposure effects; however, after adjusting the covariates, (**b**) presented that Eu made an important contribution in the mixture exposure.

The single-exposure effect is shown in Figure 3. We did not find a significant single-exposure effect in the crude model; however, after involving the covariates, the adjusted model showed a significant single-exposure effect for Eu. Compared with the other 14 REEs, the lower and the upper limit values of Eu were all more than zero in Figure 3b. It indicated that the hair concentration of Eu had a distinct association with passive smoking independent of the other 14 REEs, which might mean that Eu had a significant contribution to the overall increased risk effect of 15 REEs increasing caused by secondhand smoke.

## 4. Discussion

In this study, we investigated the association between 15 hair REEs’ concentrations and passive smoking among rural housewives in Shanxi Province of northern China. A total of 30.4% of the recruited housewives were affected by passive smoking. We found that, compared to the controls, housewives exposed to passive smoking had higher concentrations of 15 REEs. Except for Sm, the univariate and adjusted logistic models indicated higher levels of the other 14 REEs in the housewives exposed to passive smoking. The increasing linear trend of adjusted ORs of 15 REEs also strengthened their association. We further observed the overall risk of 15 REEs and the single-exposure effect of Eu in the BKMR model. In summary, our results supported the assumption that passive smoking is probably associated with elevated concentrations of hair REEs among housewives; therefore, passive smoking among housewives needs more attention.

The concentrations of REEs in hair in our study are several times lower than those in the hair of rare earth miners or people living near mining areas [15,27,28]; hence, our study is applicable to explore the association between REEs and passive smoking in the general population. Tobacco smoke is considered to contain more than 5000 substances [29], and some studies have focused on the association between REEs and tobacco smoke. A German study reported that the concentrations of Ce and La were higher in indoor air with high environmental tobacco smoke [20], which indicated that secondhand smoke might be a latent exposure source of REEs. Moreover, a cross-sectional study showed that REEs were detected in the serum of active cigarette smokers, especially La and Ce (the medians were both 0.14 ng/mL); however, REEs were not detected in its nonsmoking people [21]. These two studies suggested that Ce and La might be potential markers of tobacco smoke. Zumbado et al. further explored 32 elements of tobacco and rolling paper with ICP–MS and demonstrated that cigarette consumption was an additional source of exposure to REEs [19]. Nevertheless, we did not acquire any other reports about REEs’ concentrations among people with passive smoking, and our study found an association between REEs and passive smoking among rural women for the first time, which had similar results to the above studies. We found that REEs were significantly associated with passive smoking, and their associations were analogous; in particular, Eu might be considered a potential marker of passive smoking in rural housewives. It is noteworthy that our detection rates of 15 REEs among women with passive smoking were close to 100% and higher than the study mentioned above, which could be explained that people with passive smoking were exposed to more pollutants than those with active smoking. Compared with serum specimens, hair used in our study can reflect REEs’ chronic exposure in a certain period.

Our study had three limitations. Owing to the relatively weak causal reference of the cross-sectional study, we cannot probe the causal relationship between REEs and passive smoking. Consequently, more direct epidemiological evidence is needed in the future. In addition, our subjects were mainly of Han nationality, which might hinder the extrapolation of the conclusion to other ethnicities. In fact, nicotine and cotinine as biomarkers of passive smoking could be more objective than questionnaires. We believed that later studies could be better scientific research after these study limitations improved.

However, our study also had several strengths. First, we analyzed 15 REEs’ concentrations in hair, which could be a marker of chronic REEs’ exposure. Second, the exposure scenes of the involved subjects were less polluted and did not change greatly in the past 10 years, which can reduce the latent significant impact of other pollution. Third, the questionnaire was collected by uniformly trained healthcare workers for quality control and assurance. Fourth, we used BMKR models to find that there was a cumulative effect between hair REEs and passive smoking, and Eu might be an important marker, which enhanced the results of logistic regression model.

To our knowledge, this is the first study to examine REEs among women under passive smoking. We found that passive smoking was associated with 14 REEs except for Sm and had a cumulative effect with 15 REEs among rural housewives, and hair Eu might be a potential biomarker of secondhand smoke. Further epidemiological evidence is needed in the future.

## 5. Conclusions

This study assessed the status of passive smoking and detected the concentrations of hair REEs with ICP–MS among women in Shanxi Province of northern China. We deemed passive smoking associated with increased exposure to REEs among rural housewives, and Eu might be a potential marker of secondhand smoke exposure. Passive smoking is a common exposure for women; therefore, more measures should be taken to prevent them from secondhand smoke to avoid the prospective health risk caused by REEs.

## Figures and Tables

**Table 1:** Characteristics of included subjects.

Characteristics	Exposed Group (*n* = 117)	Control Group (*n* = 268)	*p*
	No. (*n*%)	No. (*n*%)	
Age (years) ^a^	51.88 ± 10.58	52.99 ± 10.35	0.337
BMI (kg/m^2^) ^a^	24.86 ± 3.21	24.91 ± 2.94	0.879
Occupation			
Farmer	95 (81.2)	205 (76.5)	
Nonfarmer	22 (18.8)	63 (23.5)	0.374
Education			
Junior high school or lower, or unknown	54 (46.2)	147 (54.9)	
High school or higher	63 (53.8)	121 (45.1)	0.144
Drinking wine (>50 g) (times/week)			
Never	106 (90.6)	256 (95.5)	
>1	11 (9.4)	12 (4.5)	0.101
Weak drinks (>50 mL) (times/week)			
Never	110 (94.0)	261 (97.4)	
Ever	7 (6.0)	7 (2.6)	0.184
Physical activity (times/week)			
Never	60 (51.3)	192 (71.6)	
1–3	45 (38.5)	56 (20.9)	
>3	12 (10.3)	20 (7.5)	<0.001
Menopause status			
Yes	67 (57.3)	155 (57.8)	
No	50 (42.7)	113 (42.2)	1.000

Abbreviations: BMI, body mass index. Values for certain characteristics may not be equal to 100 because of rounding. ^a^ Mean ± standard deviation shown for continuous variables.

**Table 2:** Concentrations (ng/g hair) of the fifteen rare earth elements (REEs) in the exposed group and control group.

REEs	Exposed Group (*n* = 117)		Control Group (*n* = 268)		*p*
	DR (%)	Median [IQR]	DR (%)	Median [IQR]	
La	100.00	15.21 [8.50, 22.54]	100.00	11.04 [6.11, 19.13]	0.008
Ce	100.00	30.67 [15.75, 45.96]	100.00	23.18 [13.69, 37.95]	0.015
Pr	100.00	3.55 [1.89, 5.35]	100.00	2.66 [1.58, 4.43]	0.010
Nd	100.00	12.56 [6.72, 18.69]	100.00	9.11 [5.05, 16.33]	0.007
Sm	99.15	2.29 [1.18, 3.49]	99.25	1.64 [0.94, 2.95]	0.036
Eu	99.15	1.06 [0.72, 1.55]	99.25	0.90 [0.57, 1.29]	0.006
Gd	99.15	3.27 [1.52, 4.57]	99.25	2.23 [1.24, 3.80]	0.010
Tb	99.15	0.35 [0.19, 0.56]	99.25	0.24 [0.14, 0.49]	0.008
Dy	99.15	1.87 [1.03, 3.20]	99.25	1.44 [0.82, 2.74]	0.041
Ho	99.15	0.33 [0.18, 0.56]	99.25	0.24 [0.13, 0.46]	0.026
Er	99.15	1.04 [0.51, 1.60]	99.25	0.78 [0.43, 1.35]	0.020
Tm	99.15	0.14 [0.08, 0.22]	99.25	0.10 [0.06, 0.19]	0.013
Yb	99.15	0.92 [0.54, 1.44]	99.25	0.62 [0.37, 1.21]	0.002
Lu	99.15	0.15 [0.09, 0.23]	99.25	0.10 [0.06, 0.20]	0.003
Y	99.15	9.21 [5.37, 14.19]	99.25	7.23 [4.31, 12.70]	0.033

Abbreviations: DR, detection rate; IQR, interquartile range.

**Table 3:** Logistic regression models for binary variables of 15 REEs.

REEs ^a^	Univariate Model		Adjusted Model ^b^	
	OR	95% CI	OR	95% CI
La	1.69	1.09–2.63	1.68	1.06–2.67
Ce	1.88	1.21–2.92	1.90	1.19–3.03
Pr	1.78	1.15–2.77	1.79	1.12–2.84
Nd	2.30	1.47–3.61	2.40	1.49–3.86
Sm	1.53	0.99–2.38	1.52	0.96–2.41
Eu	1.69	1.09–2.63	1.72	1.09–2.72
Gd	2.19	1.40–3.42	2.24	1.40–3.60
Tb	1.69	1.09–2.63	1.68	1.06–2.66
Dy	1.78	1.15–2.77	1.83	1.15–2.91
Ho	1.78	1.15–2.77	1.89	1.18–3.02
Er	1.78	1.15–2.77	1.81	1.14–2.87
Tm	1.78	1.15–2.77	1.82	1.14–2.90
Yb	2.30	1.47–3.61	2.36	1.47–3.78
Lu	1.88	1.21–2.92	1.86	1.17–2.97
Y	1.69	1.09–2.63	1.71	1.08–2.71

Abbreviations: OR, odds ratio; CI, confidence interval. ^a^ REEs are bisected according to the median (P_50_). ^b^ The covariates include age, BMI, occupation, education, drinking wine, weak drinks, physical activity, and menopause status.

## Data Availability

The data are available in the main text or the Appendix A, or can be obtained by contacting the correspondence authors (Z.L. and N.L.).
